# Time Course of Placebo Effect of Acupuncture on Pain: A Systematic Review

**DOI:** 10.1155/2013/204108

**Published:** 2012-11-28

**Authors:** Yun Hyung Koog, Won Young Jung

**Affiliations:** ^1^Honam Research Center, Medifarm Hospital, Suncheon, Republic of Korea; ^2^Department of Oriental Medicine, Medifarm Hospital, Suncheon, Republic of Korea; ^3^Department of Neurology, Medifarm Hospital, Suncheon, Republic of Korea

## Abstract

*Objectives*. Our objective was to investigate the time course of the placebo effect of acupuncture on pain and the factors affecting the placebo effect. *Methods*. Previously we retrieved three-armed randomized acupuncture trials including sham and no-treatment groups which were published until October 2009. We searched electronic databases again to identify additional trials from October 2009 to December 2011. After a screening of trials, fifteen three-armed acupuncture trials for pain were included in the analysis. Standardized mean differences between the sham and no-treatment groups were calculated for placebo effect. We then plotted the magnitude of the placebo effect over time. *Results*. The placebo effect gradually has increased for 12 weeks with a standardized mean difference of 0.74 (95% CI: 0.54 to 0.94). Then it decreased after 12 weeks as time passed. When the placebo effects were compared for factors including methodological qualities, they were not affected by all factors, except patient blinding. Trials with sufficient patient blinding showed a larger placebo effect at 8 weeks than those with insufficient patient blinding (*P* = 0.0009). *Conclusion*. The placebo effect of acupuncture showed a unique pattern, which was affected by insufficient patient blinding.

## 1. Introduction

Randomized trials are necessary to evaluate the effectiveness of acupuncture because acupuncture provides inconsistent benefits to patients who are randomly allocated to acupuncture treatment compared to those who directly choose [1]. Randomized trials can provide unbiased results about the efficacy of acupuncture if acupuncture is controlled with a sham acupuncture. For this aim, many efforts have been made to develop optimal sham acupuncture. In fact, sham acupunctures have changed from normal needling of nonacupoints to minimal needling of nonacupoints and nonpenetrating stimulation [2].

Nevertheless, it has been reported that all types of sham acupuncture may not be physiologically inert [3–5]. Moreover, according to a series of recent studies [6, 7], there is a high possibility of publication bias, where trials with negative results in a treatment group would be less likely to be published, in the three-armed randomized trials including sham and no-treatment groups. When the bias was considered, the magnitude of the placebo effect was calculated to be large [6].

However, all these findings [3–7] should be interpreted with caution because they were based on the data measured at one time point, as specified by authors of trials. To date, it has not been studied how the placebo effect changes over time. Therefore, we attempted to investigate a one-year time course of the placebo effect of acupuncture. Because bias could be introduced by combining results of trials from different conditions [8], we focused on trials that treated pain. 

## 2. Method

### 2.1. Study Selection

Previously, 32 three-armed randomized acupuncture trials were identified using MEDLINE, CINAHL, EMBASE, and the Cochrane Registered Trials from their launch through October 2009 [6, 7]. From October 2009 to December 2011, the first author searched MEDLINE, SCOPUS, and the Cochrane Registered Trials using the same terms “acupuncture,” “electroacupuncture,” and “electro-acupuncture.” Then the first author selected randomized clinical trials that met the following conditions: (1) reported data on pain, (2) included an acupuncture group where dry needles were inserted in traditional or painful points, (3) included a sham group where an intervention was considered a sham or a placebo acupuncture in the text, (4) included a no-treatment group where no treatment was applied, and (5) compared the above three groups under identical conditions in one trial. Trials conducted for only one day were excluded. 

### 2.2. Data Extraction

Since the trial duration and assessment time points varied across trials, the assessment time points were grouped into 6 time windows: 0 week, 4 weeks (>0 and ≤4), 8 weeks (>4 and ≤8), 12 weeks (>8 and ≤12), 16 weeks (>12 and ≤16), and 52 weeks (>16 and ≤52). The first author then extracted end-point data on pain intensity (e.g., visual analogue scales or other ranking scales) for sham and no-treatment groups reported in those time windows. When more than one pain outcome measure was reported, visual analogue scales were preferred. If end-point data were not available, data on changes from baseline were used. If no data were available, previous studies [9, 10] were referenced. Information on trial characteristics, including the methodological qualities, was also extracted. All data were verified by the second author.

### 2.3. Data Analysis

For each time window, the standardized mean differences (SMDs) were calculated using the sham and no-treatment groups to assess the placebo effect. To analyze data from all time windows similarly, the random effects model was used to present summary estimates [11]. The *I* ^2^ test was conducted to measure the heterogeneity within each time window [12]. *I* ^2^ values of 25, 50, and 75% are referred to as low, moderate, and high inconsistencies between the trials, respectively. The multivariate analysis was also performed to confirm that the results were robust [13]. Because within-study correlations are unknown, the “riley” option was used.

In the secondary analysis, the following items were examined: allocation concealment, patient blinding, intention-to-treat analysis, standardized cointerventions, additional medical help (e.g., the use of rescue analgesics), sham acupuncture type, patient-therapist interaction, the number of treatment sessions, and the number of needles per session. Allocation concealment was considered adequate if researchers screening patients could not predict the next treatment for a patient. Patient blinding was considered adequate if patients could not notice the treatment they receive. Specifically, to determine the status of patient blinding, we focused on the patients' real guesses of treatment credibility. Because analytical methods varied across trials and the original data were insufficiently detailed in trials, we extracted the number of patients who perceived to have received true acupuncture in the acupuncture and sham groups and calculated the significance using the two sample proportion test. Intention-to-treat analysis was considered adequate if all patients assessed at baseline were included in the analysis. All items were analyzed on a dichotomous basis. For the number of treatment sessions and needles per session, a median value was used as a cutoff point. 

STATA 11.0 was used for the analyses. The data are presented as the SMDs with a 95% confidence interval, where a positive SMD indicates that sham acupuncture was more effective than no-treatment. The significance was assessed at the level of 0.05. For the secondary analyses, the SMDs at each time point were compared at the level of 0.008 using the interaction test [14] followed by the Bonferroni correction. 

## 3. Results

**Figure 1:**
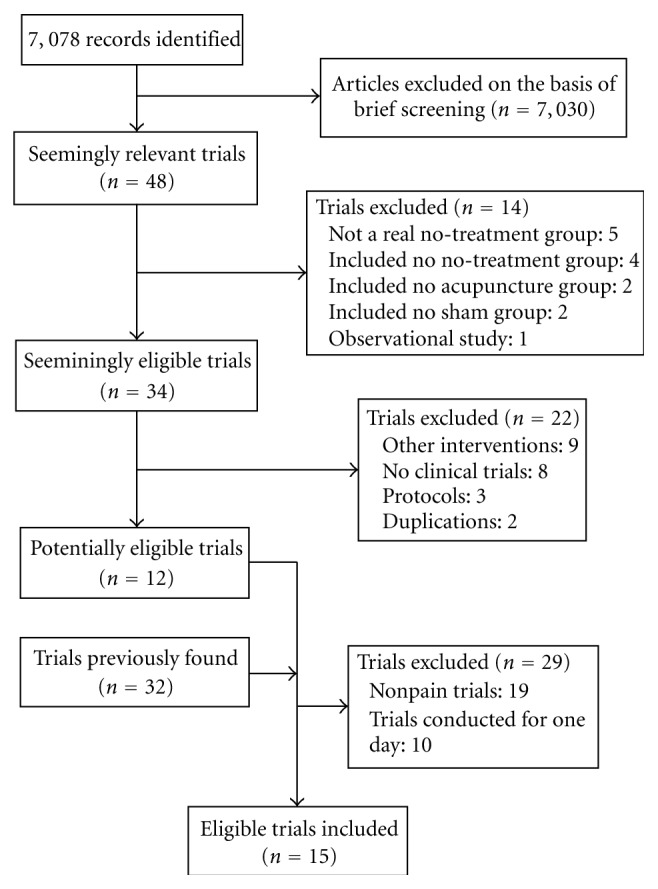
Study flow diagram.

Among 7078 citations (MEDLINE 546, SCOPUS 5804, and the Cochrane Registered Trials 728) in a new search, we identified 12 potentially eligible trials (Figure 1). After a screening of 44 trials including 32 trials that were analyzed in previous studies [6, 7], we further excluded 29 trials: 19 reported on non-pain-related diseases, and ten were conducted for one day. In total, 15 trials that met the selection criteria were analyzed [15–29].

### 3.1. Description of the Included Trials

Table 1 shows the characteristics of the included trials. The most frequently studied conditions were low back pain [16, 20, 21, 23], followed by knee osteoarthritis [15, 17, 29]. Regarding sham type, ten trials used a needle inserted superficially at nonacupoints [15–21, 25, 28, 29], two trials used a non-penetrating acupuncture-like instrument [22, 23], two used a needle inserted normally at nonacupoints [24, 26], and one used a disconnected laser instrument [27]. Most of the trials concealed allocation adequately, while three trials did not [25–27]. Six trials reported sufficient patient blinding [15, 17, 19, 23, 28, 29], but the significance altered (*P* = 0.02) in one trial [19] according to our reanalysis. The median treatment duration and the median number of treatment sessions were six weeks and twelve, respectively. In total, 2591 patients were included at baseline: 1421 in the sham group and 1170 in the no-treatment group.

### 3.2. Time Course of Placebo Effect

**Figure 2:**
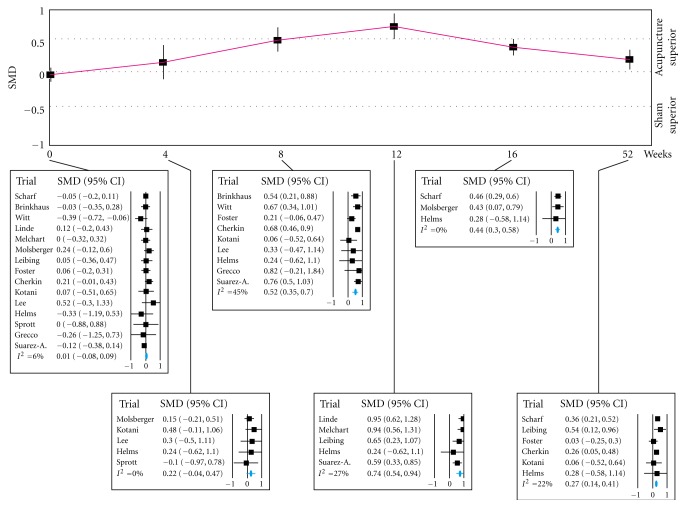
Time course of the placebo effect. SMD: standardized mean difference.

Figure 2 presents summary estimates of the placebo effect. The placebo effect gradually increased from baseline to 12 weeks, with an SMD of 0.74 (95% CI: 0.54 to 0.94). Then, it gradually decreased to 0.27 (0.14 to 0.41) at 52 weeks. The degree of the heterogeneity was low to moderate at all time points. When the multivariate analysis was conducted, we failed in obtaining the result.

**Figure 3:**
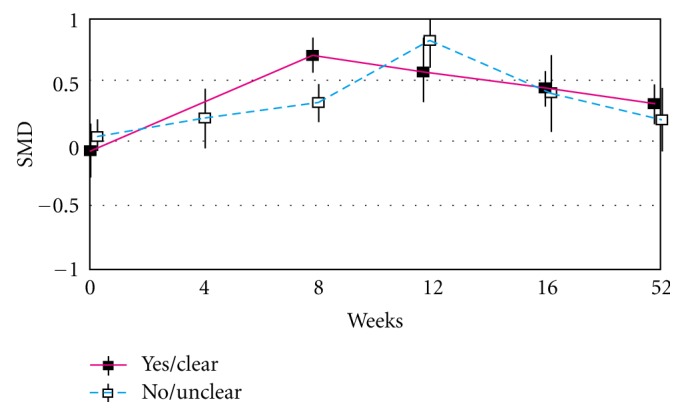
Time course of the placebo effect based on patient blinding. SMD: standardized mean difference.

When the placebo effects were compared for factors including methodological qualities, they were not affected by all factors, except patient blinding. For patient blinding, the patterns were significantly different at 8 weeks (Figure 3). SMDs for sufficient patient blinding and insufficient patient blinding were 0.71 (0.56 to 0.86) and 0.30 (0.12 to 0.49) at 8 weeks (*P* = 0.0009), respectively.

## 4. Discussion

We investigated the pattern of the placebo effect of acupuncture over time, using three-armed trials for pain. The placebo effect gradually increased from baseline until 12 weeks, and decreased after 12 weeks as time passed. When the placebo effects were compared for factors including methodological qualities, they were not affected by all factors, except patient blinding. Trials with sufficient patient blinding showed a larger placebo effect at 8 weeks than those with insufficient patient blinding (*P* = 0.0009). 

Because we examined the placebo effect over time by separately combining the data for each time window, our findings are highly informative in understanding the dynamic pattern of the placebo effect. However, although this approach has been used in recent studies [30, 31], there are several limitations. First, not all of the trials provided data relating to each time window. Because of the sparsity of data available for each time window, possible correlations between results for the different time windows were not addressed. Therefore, we could not determine the robustness of our results. Second, other time windows are possible to depict the placebo effect. For example, the placebo effect can be described over three time windows, with SMDs of 0.35 (0.18 to 0.51) in 0 to 6 weeks, 0.66 (0.52 to 0.80) in 6 to 12 weeks, and 0.29 (0.17 to 0.41) in 12 to 52 weeks. The degree of the heterogeneity was ≤27%. Although this example shows a unique pattern, the placebo effect also increased from baseline until 12 weeks, as with the current result. 

Numerous studies [6, 7, 9, 32] have investigated the placebo effect of acupuncture compared with a no-treatment group. They analyzed data at one time point and interpreted findings in terms of two aspects: (1) the magnitude of the placebo effect of acupuncture and (2) the efficacy of acupuncture compared with sham acupuncture. 

First, two previous studies [9, 32] demonstrated that the high degree of variability associated with the placebo effect led to cases where sham acupuncture was effective in some situations and ineffective in others. However, they found in general the large placebo effect on pain, with an SMD of 0.42 (0.23 to 0.60) [9] and of 0.53 (0.39 to 0.67) [32], respectively. Specifically, one study [9] argued that this large placebo effect may be associated with response bias, where patients report better outcomes to please researchers. Meanwhile, recent two studies argued that the variable placebo effect may be due to the result of the natural process of publication in journals [6, 7]. When this publication bias was considered, the SMD for the placebo effect was estimated to be 0.44 (0.29 to 0.59) in general conditions [6].

However, according to the current findings, the placebo effect is the most prominent at 12 weeks, with an SMD of 0.74 (0.54 to 0.94). Although we analyzed the similar set of trials to that of previous study [9], this SMD is much greater than that of previous studies [9, 32]. Because previous studies analyzed the data measured at one time point [6, 7, 9, 32], their analyses may be confounded by measurement time. In fact, when we used a median of 6 weeks as a cutoff point and reanalyzed the data of one study [9], the summary estimates were different (*P* = 0.002): SMD of 0.32 (0.16 to 0.49) for 0 to 6 weeks and 0.65 (0.52 to 0.78) for 8 to 12 weeks. This was also true when reanalyzing the data from another study [32], in which the summary estimates were different (*P* < 0.001): SMD of 0.09 (−0.12 to 0.30) for 0 to 6 weeks and 0.64 (0.49 to 0.79) for 8 to 12 weeks. 

The current findings also show that the sham acupuncture was effective at 52 weeks. It can be argued that because sham acupunctures may not be physiologically inert [3–5], they may have affected the long-term pain outcomes. However, considering that they have been developed to reduce the specific effect of acupuncture, this is not the case. There is also evidence that the placebo effect may be maintained over 52 weeks in the more extreme cases (e.g., surgery) [33]. It is unclear at present whether the placebo effect of acupuncture lasts over 52 weeks. Therefore, we believe that the substantial magnitude (maybe ≤0.27) of the placebo effect at 52 weeks appears to be related to a response bias. This finding supports other study [34] arguing that the response bias is a major problem in estimating the exact placebo effect.

Second, previous studies showed that the efficacy of acupuncture compared with sham acupuncture was small in magnitude [6, 9, 32]. However, this finding was interpreted differently among studies. One study argued that the efficacy of acupuncture might not be clinically relevant [9]. Meanwhile, other study argued that since the total effect of acupuncture including specific and nonspecific effect appeared to be moderate in magnitude, acupuncture could be clinically useful from a pragmatic decision maker's viewpoint [32]. They all argued that insufficient patient blinding may be problematic in estimating the exact efficacy of acupuncture, but none of these studies presented the evidence.

Meanwhile, we did not focus on the acupuncture and sham acupuncture because there is a great controversy surrounding placebos [35]. Nonetheless, our finding suggests that measurement time is one of important factors to study the efficacy of acupuncture in future. In a clinical practice, clinicians use acupunctures with a variety of skills. Thus, all types of acupuncture do not show the greatest efficacy at the same time point. However, there are few studies commenting on why they assessed the efficacy at the designated time point. If we know about the effect pattern for acupunctures tested in trials, we can show the maximized efficacy of the acupunctures. 

In addition, our finding emphasizes the importance of patient blinding. Insufficient patient blinding underrated the magnitude of the placebo effect at 8 weeks. For this reason, the efficacy of acupuncture may be exaggerated at 8 weeks in trials with insufficient patient blinding. There are two possible reasons of why this finding was not identified in previous studies [6, 7, 9, 32]. First, previous studies simultaneously analyzed the data at different time points, while we categorized the data based on measurement time. Second, we reanalyzed the trials to check whether patient blinding was sufficient or not. Perhaps, a combination of two reasons might induce this discrepancy.

In conclusion, the placebo effect of acupuncture showed a unique pattern according to time: The placebo effect gradually increased from baseline until 12 weeks and decreased after 12 weeks as time passed. This pattern was affected by patient blinding. The analgesic effect of sham acupuncture was the most prominent at 12 weeks. We believe that our findings can assist researchers in the design and conduct of acupuncture trials for pain.

## Figures and Tables

**Table 1:** Characteristics of included trials.

Study	Clinical conditions	Number of randomized patients∗	Allocation concealment	Treatment duration (session)	Sham type
Scharf et al. [15]	Knee osteoarthritis	709 (367, 342)	Central randomization	6 weeks (10^†^)	Superficial needling at nonacupoints
Brinkhaus et al. [16]	Low back pain	154 (75, 79)	Centralized telephone randomization	8 weeks (12)	Superficial needling at nonacupoints
Witt et al. [17]	Knee osteoarthritis	150 (76, 74)	Centralized telephone randomization	8 weeks (12)	Superficial needling at nonacupoints
Linde et al. [18]	Migraine	157 (81, 76)	Centralized telephone randomization	8 weeks (12)	Superficial needling at nonacupoints
Melchart et al. [19]	Tension-type headache	138 (63, 75)	Centralized telephone randomization	8 weeks (12)	Superficial needling at nonacupoints
Molsberger et al. [20]	Low back pain	121 (61, 60)	Central telephone randomization	4 weeks (12)	Superficial needling at nonacupoints
Leibing et al. [21]	Low back pain	100 (50, 50)	Unclear	12 weeks (20)	Superficial needling at nonacupoints
Foster et al. [22]	Knee osteoarthritis	235 (119, 116)	Centralized telephone randomization	3 weeks (6)	Nonpenetrating needles at acupoints
Cherkin et al. [23]	Low back pain	323 (162, 161)	Central randomization	7 weeks (10)	Toothpick at acupoints
Kotani et al. [24]	Abdominal scar pain	46 (23, 23)	Sequentially sealed opaque envelopes	4 weeks (20)	Normal needling at nonpainful points
S. H. Lee and B.C. Lee [25]	Prostatitis	26 (13, 13)	Unclear	6 weeks (12)	Superficial needling at nonacupoints and mock electrical stimulation
Helms [26]	Primary dysmenorrhea	22 (11, 11)	Unclear	12 weeks (9)	Normal needling at nonacupoints
Sprott [27]	Fibromyalgia	20 (10, 10)	Unclear	3 weeks (6)	Disconnected laser instrument
Greco et al. [28]	Systemic lupus erythematosus	16 (8, 8)	Sequentially sealed opaque envelopes	5 weeks (10)	Superficial needling at nonacupoints
Suarez-Almazor et al. [29]	Knee osteoarthritis	374 (302, 72)	Sequentially sealed opaque envelopes	6 weeks (12)	Superficial needling at nonacupoints

^*^Values are for total randomized patients (patients in sham and no-treatment groups, resp.).

^†^This trial provided additional sessions for patients who experienced a pain reduction.
